# PD-(L)1 Inhibitors as Monotherapy for the First-Line Treatment of Non-Small-Cell Lung Cancer Patients with High PD-L1 Expression: A Network Meta-Analysis

**DOI:** 10.3390/jcm10071365

**Published:** 2021-03-26

**Authors:** Margarita Majem, Manuel Cobo, Dolores Isla, Diego Marquez-Medina, Delvys Rodriguez-Abreu, Joaquín Casal-Rubio, Teresa Moran-Bueno, Reyes Bernabé-Caro, Diego Pérez-Parente, Pedro Ruiz-Gracia, Marta Marina Arroyo, Luis Paz-Ares

**Affiliations:** 1 Medical Oncology, Hospital de la Santa Creu i Sant Pau, 08041 Barcelona, Spain; 2 Medical Oncology, Hospital Regional Universitario de Málaga, 29010 Málaga, Spain; manuelcobodols@yahoo.es; 3 Medical Oncology, University Hospital Clínico Lozano Blesa, Zaragoza, 50009 IIS Aragón, Spain; lola.isla@gmail.com; 4 Medical Oncology, University Hospital Miguel Servet, 50009 Zaragoza, Spain; dmarmed@hotmail.com; 5 Medical Oncology, Hospital Universitario Insular de Gran Canaria, 35016 Las Palmas de Gran Canaria, Spain; delvysra@yahoo.com; 6 Medical Oncology Service, Hospital Álvaro Cunqueiro, 36213 Vigo, Spain; joaquin.casal.rubio@sergas.es; 7 Medical Oncology, Hospital Universitari Germans Trias i Pujol, 08916 Badalona, Spain; mmoran@iconcologia.net; 8 Medical Oncology Department, Hospital Virgen del Rocío, 41013 Seville, Spain; reyesbernab@yahoo.es; 9 Medical Affairs Department, Roche Farma S.A, 28042 Madrid, Spain; diego.perez@roche.com (D.P.-P.); pedro.ruiz.pr1@roche.com (P.R.-G.); marta.marina@contractors.roche.com (M.M.A.); 10 Medical Oncology, Hospital 12 de Octubre, 28041 Madrid, Spain; lpazaresr@seom.org

**Keywords:** non-small cell lung cancer, network meta-analysis, immunotherapy, first-line treatment, PD-(L)1 inhibitors, efficacy

## Abstract

Programmed cell death-ligand 1 (PD-L1) has emerged as a potential biomarker for selection of patients more likely to respond to immunotherapy and as a prognostic factor in non-small cell lung cancer (NSCLC). In this network meta-analysis, we aimed to evaluate the efficacy of first-line anti-PD-(L)1 monotherapy in advanced NSCLC patients with high PD-L1 expression (≥50%) compared to platinum-based chemotherapy. We also evaluated efficacy outcomes according to tumor mutational burden (TMB). To that end, we conducted a systematic review. Six clinical trials with 2111 patients were included. In head-to-head comparisons, immunotherapy showed a significant improvement in progression-free survival (PFS: HR_pooled_ = 0.69, 95% CI: 0.52–0.90, *p* = 0.007), overall survival (OS: HR_pooled_ = 0.69, 95% CI: 0.61–0.78; *p* < 0.001) and overall response rate (ORR) (Risk ratio (RR)_pooled_ = 1.354, 95% CI: 1.04–1.762, *p* = 0.024). In the assessment of relative efficacy for PFS through indirect comparisons, pembrolizumab (results from KEYNOTE-024) ranked highest followed by cemiplimab and atezolizumab, with statistical significance determined for some of the drugs. In terms of OS, cemiplimab ranked highest followed by atezolizumab and pembrolizumab, although non-significant OS was determined for these drugs. In conclusion, PD-(L)1 inhibitor monotherapy improves efficacy outcomes in the first line setting of advanced NSCLC patients with high PD-L1 expression. Evaluations with longer follow up are still needed to determine the superiority of any specific drug.

## 1. Introduction

Lung cancer is the leading cause of cancer death among men, and the second among women worldwide [1]. Non-small cell lung cancer (NSCLC), the most frequent lung carcinoma, accounts for 85% of all diagnosed cases [2] and is frequently detected in the advanced stages [3]. Its prognosis is poor, with five-year survival rates of 0–5% with the use of chemotherapy [4], which has been the only systemic therapeutic strategy available for decades [3]. Since then, the understanding of the biology of this cancer has rapidly increased and the use of targeted therapy with tyrosine kinase inhibitors (TKIs) improve the management of patients with oncogenic driven cancers and their survival rates. Major progress was made with the emergence of the immunotherapy with reduced overall toxicity and almost complete absence of non-specific side effects compared to chemotherapy and other classic cancer therapies, but with specific toxicity profiles depending on the mechanisms of action [5,6,7]. In this regard, immunotherapy targeting programmed cell death-1 (PD-1) and programmed cell death-ligand 1 (PD-L1) has markedly improved the overall survival (OS) of patients, not only in those with metastatic NSCLC, but also in patients with locally advanced disease and extensive-stage small-cell lung cancer [8,9,10,11,12,13].

PD-L1 is expressed on tumor cells (TCs) and tumor-infiltrating immune cells (ICs) [14]. The binding of PD-L1 to its receptor PD-1 on activated T cells can lower the T-cell immune responses and prevent elimination of tumor cells [15,16]. In addition to its central role as a key element of current immunotherapy strategies, PD-L1 has emerged as a potential prognostic factor and biomarker to predict which patients are more likely to respond to immunotherapy in NSCLC [17,18,19,20,21,22,23,24,25,26]. The success of the anti-PD-1 antibodies, nivolumab and pembrolizumab, and the PD-L1 antibody, atezolizumab, approved so far in patients with previously treated NSCLC [27,28,29] understandably aroused considerable interest in extending these therapies to the first-line setting, both in combination with chemotherapy regardless of PD-L1 expression [30], and in monotherapy in PD-L1-positive patients. In this context, different cut-off values for PD-L1 expression were used in clinical trials evaluating PD-(L)1 inhibitors as monotherapy vs. chemotherapy in patients with no targetable mutations. In the phase III open-label KEYNOTE-024 trial [31], metastatic NSCLC tumors with a PD-L1 tumor proportion score (TPS) ≥50% showed improved progression-free survival (PFS) (Hazard ratio (HR) 0.50; 95% confidence interval (CI) 0.37–0.68), *p* < 0.001), and overall response rate (ORR) (44.8% vs. 27.8%) with pembrolizumab. Furthermore, at the most recent follow-up analysis (median time from randomization to data cut-off was 59.9 (55.1–68.4) months), median OS also improved: 26.3 months with pembrolizumab vs. 13.4 months with chemotherapy (HR 0.62; 95% CI 0.48‒0.81) [32]. These results were confirmed in a subsequent evaluation of pembrolizumab in the phase III open-label KEYNOTE-042 study [33], in which OS improved with the PD-1 antibody compared with chemotherapy (HR 0.69; 95% CI 0.56–0.85, *p* = 0.0003); this was also observed at other PD-L1 TPS cut-offs (TPS ≥ 20% and TPS ≥ 1%). Median PFS was 7.1 months (95% CI 5.9–9.0) in the pembrolizumab group and 6.4 months (95% CI 6.1–6.9) in the chemotherapy group. In the case of atezolizumab, a recent interim analysis of the phase III IMpower110 trial [34] has recently shown a statistically significant and clinically meaningful improvement in OS vs. platinum-based chemotherapy in a PD-L1–high population (20.2 months vs.13.1 months; HR, 0.59; 95% CI: 0.40, 0.89, *p* = 0.0106), as well as longer PFS (8.1 months vs. 5 months; HR, 0.63; 95% CI: 0.45, 0.88, *p* = 0.0007 [34]. Unlike atezolizumab and pembrolizumab, neither nivolumab nor durvalumab demonstrated statistically significant survival benefits in previously untreated PD-L1-positive mNSCLC (CheckMate 026 [35] and MYSTIC [36] trials, respectively). Finally, cemiplimab, a highly potent anti-PD-1 already approved for the treatment of advanced cutaneous squamous cell carcinoma (CSCC), is being evaluated in monotherapy vs. investigator’s choice platinum-doublet chemotherapy in patients with advanced NSCLC and PD-L1 TPS ≥50% (EMPOWER Lung-01 trial [37]). Interim results (median follow-up: 10 months) have shown that cemiplimab monotherapy significantly improves PFS and OS vs. chemotherapy in patients with high PD-L1 expression (PFS: 8.2 months vs. 5.7 months; HR, 0.54; 95% CI: 0.43, 0.68, *p* < 0.0001). Median OS was not reached for the cemiplimab arm vs. 14.2 months for the control arm; HR, 0.57; 95% CI: 0.42, 0.77, *p* = 0.0002). 

The literature suggests that the first-line immunotherapy monotherapy strategy has become the new standard of care in locally advanced and metastatic NSCLC patients with high PD-L1 expression levels and no targetable mutations. Nevertheless, the because of the lack of direct cross-comparison studies or comparisons between trials, choosing the best treatment is still challenging. Apart from PD-L1, the tumor mutational burden (TMB) has recently emerged as a promising biomarker for immune checkpoint inhibitor (ICI) patient stratification [38]. TMB is defined as the total number of non-synonymous mutations per coding area of a tumor genome and is an indirect measure of tumor-derived neoantigens [39,40]. Several TMB testing panels are currently available, and their variability needs to be fully understood. Additionally, optimal TMB cut-offs for treatment decisions may need to be specified across different cancer types [41]. In NSCLC, preliminary results support this potential predictive role for TMB [38,42], but more evidence is needed. Thus, several clinical trials have assessed the predictive value of TMB in different studies with combined ICI regimens, such as nivolumab plus ipilimumab [43,44,45,46], or ICI monotherapy, such as with atezolizumab [47,48,49] and pembrolizumab [50].

The aim of this study was to conduct a network meta-analysis (NMA) to evaluate the efficacy of the available PD-(L)1-containing immunotherapy strategies in monotherapy for the first-line treatment of patients with high PD-L1 expression (≥50%) and locally advanced or metastatic NSCLC. We also evaluated efficacy outcomes according to TMB. 

## 2. Materials and Methods

### 2.1. Search Strategies and Study Selection

We conducted a systematic search in PubMed to identify all eligible trials from inception until 1 November 2020, with no start date limit applied. Literature search terms used were “non-small cell lung cancer” (or “NSCLC”), “PD-L1”, “PD-1”, “pembrolizumab”, “nivolumab”, “atezolizumab”, “durvalumab”, “cemiplimab”, and all terms related to clinical trial registration (ClinicalTrials.gov, EU Clinical Trials Register, ISRCTN and ANZCTR. Accessed: 10. Dec. 2020). We also performed an additional search for abstracts presented at meetings or conferences held by the American Society of Clinical Oncology (ASCO), European Society for Medical Oncology (ESMO), American Association for Cancer Research for Medical Oncology (AACR) and World Conference on Lung Cancer (WCLC).

### 2.2. Selection Criteria

Only phase III trials conducted in patients with locally advanced/advanced NSCLC selected according to their PD-L1 expression status, not previously treated for their metastatic disease and receiving first-line PD-(L)1 monotherapy were eligible for inclusion. In order to compare homogenous populations, only subjects with PD-L1 ≥50% were considered for this NMA, and only studies reporting efficacy outcomes for PD-(L)1 monotherapy expressed as PFS or OS were included. Observational studies, editorials, reviews and commentaries were excluded. Studies conducted in subsets of patients already included in their corresponding pivotal trials were also excluded. 

### 2.3. Statistical Analysis

We performed a NMA to indirectly compare all monotherapy treatments against the common comparator, chemotherapy, an NMA was conducted. A specific application of the generalized pairwise modelling (GPM) framework [51] was applied. The Bucher method [52] was used for adjusted indirect comparisons. Cox proportional HRs along with their corresponding 95% CIs were used as the summary estimates of relative treatment effects. Summary league tables were generated for all comparisons (OS and PFS). Agents with higher efficacy appear in the first column and agents with lower efficacy compared to the first agent are presented in rows in descending order of efficacy.

For direct comparisons, the DerSimonian–Laird random effects model for main and subgroup analyses was implemented, assessing heterogeneity of effect-size estimates from the individual studies with Cochran’s Q test and the I^2^ statistic. Additionally, a meta-analysis (MA) corresponding to analysis of binary data of proportions was also performed using a DerSimonian–Laird random effects model without transformed proportion. A high level of heterogeneity was considered if I^2^ was greater than 50%. Statistical significance was reached for *p*-values less than 0.05. Analyses were not controlled for multiplicity; no alpha was assigned to the different analyses. HRs and 95% CI for OS and PFS from the overall population and subgroups from each individual trial of advanced NSCLC were calculated (only OS subgroup analysis was performed). For dichotomous data, odds ratios (OR) were estimated. The NMA was performed using Open Meta Analyst v. 10 (Center for Evidence Synthesis in Health, Brown University). Heterogeneity between studies must be considered as guidance only due to the relatively low number of trials included in this NMA [53]. Recommendations of the Cochrane Collaboration and the Preferred Reporting Items for Systematic Reviews and Meta-Analyses (PRISMA) guidelines were followed for this MA [54].

Sensitivity analyses did not quantitatively alter the results or conclusions of the main analyses.

## 3. Results

### 3.1. Studies Included in the Meta-Analysis

**Figure 1:**
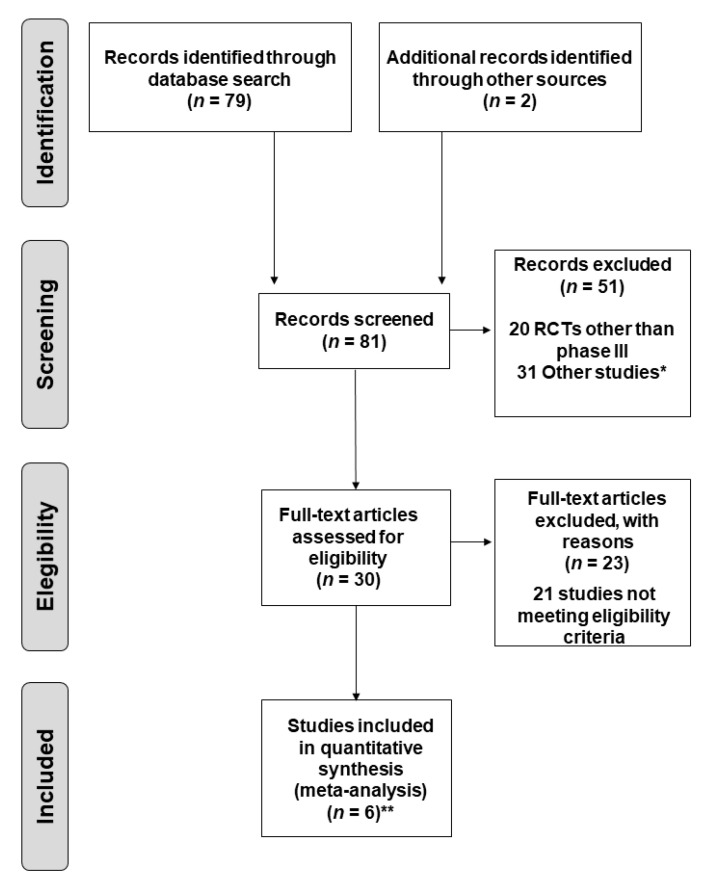
Flow chart of study selection (up to 1 November 2020). RCTs, randomized controlled trials * Other studies included pooled analyses, post-marketing studies, clinical trial protocols, patient-reported outcome assessments and any study on biomarkers/gene profiling. ** Two publications (one of them presented at the ASCO congress) were included for one of the trials (KEYNOTE-024 [10,33]).

A total of 79 records from PubMed were screened. Two additional studies presented at ASCO [13] and ESMO [37] were also included. Study selection and exclusion criteria are summarized in Figure 1. Finally, six clinical trials carried out with 2111 patients met the inclusion criteria and were included in the MA [13,31,33,34,35,36,37].

### 3.2. Study Characteristics

The specific characteristics of the studies included in the MA are summarized in Table 1. The control arm in all studies was platinum-based chemotherapy. Two key methodological differences in cemiplimab clinical trials should be noted. First, in EMPOWER Lung-01 [37], patients in the cemiplimab arm who responded to cemiplimab monotherapy were allowed to continue the drug plus treatment with four cycles of chemotherapy in the event of progressive disease. In KEYNOTE-024 [13,31], EMPOWER Lung-01 [37], and CheckMate-026 [35] crossover was permitted. Second, studies on cemiplimab did not include a never-smoker population. 

In terms of primary endpoints, PFS and OS were co-primary endpoints in both the MYSTIC [36] and EMPOWER Lung-01 [37] studies; two studies (KEYNOTE-024 and CheckMate-026) had PFS as the primary endpoint [13,31,35] while two others (IMpower-110 and KEYNOTE-042) used OS [33,37]. Final PFS data were reported in three studies included in this NMA (KEYNOTE-024 [13,31], MYSTIC [36], and CheckMate-026 [35]), while final data for OS was available for only one of them [36]. Interim analyses were provided for the other four [33,34,35,37]. Both endpoints were evaluated in the wild-type intention-to-treat (ITT) population (patients with EGFR or ALK mutations were excluded from all of the studies according to the eligibility criteria).

All the studies included patients with squamous and non-squamous disease, stratified according to their histology [13,31,33,34,35,36,37]. Additionally, all studies included metastatic patients, except for KEYNOTE-042 [33], which also included locally advanced NSCLC patients. Patient population characteristics of all the studies included in the MA are shown in Supplementary Table S1.

### 3.3. Efficacy Endpoints in the Overall Population

**Figure 2:**
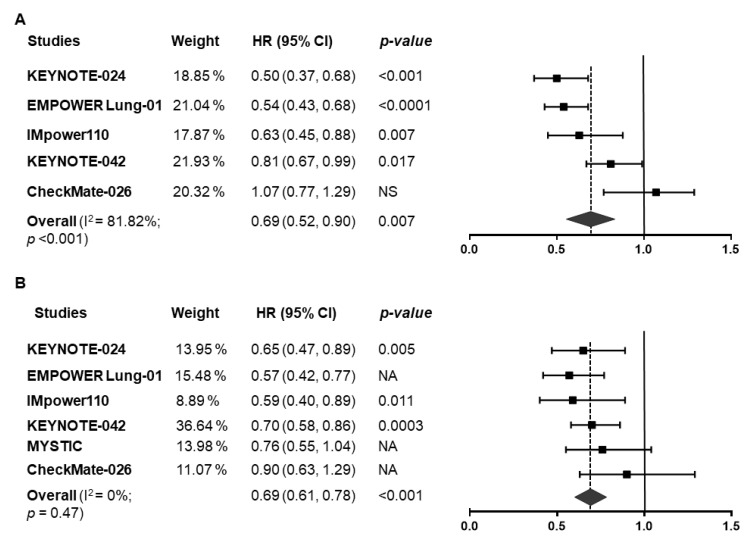
Forest plot of pooled hazard ratios for (**A**) progression-free survival (PFS) and (**B**) overall survival (OS) in patients who received PD-1/PD-L1 inhibitors vs. chemotherapy alone. HR, hazard ratio; CI, confidence interval.

The evidence formed a connected star-shaped network (Supplementary Figure S1). Median PFS ranged from 5 to 6.4 months in the control arms, and from 5.4 to 10.3 months in the treatment arms. Median OS ranged from 12.2 to 15.9 months in the control arms, and from 13.9 to 26.3 months in the treatment arms. Monotherapy with three drugs (pembrolizumab, cemiplimab and atezolizumab) showed a significant improvement in PFS compared to chemotherapy in head-to-head comparisons (PFS: HR_pooled_ = 0.69, 95% CI: 0.52–0.90, *p* = 0.007, Figure 2A). The same drugs also showed improvements in OS (OS: HR_pooled_ = 0.69, 95% CI: 0.61–0.78; *p* < 0.001, Figure 2B). The ORR also significantly improved with PD-(L)1 inhibitor monotherapy (Risk ratio (RR)_pooled_ = 1.354, 95% CI: 1.04–1.762, *p* = 0.024, Supplementary Figure S2).

In indirect comparisons of PFS (Table 2; Results were considered separately for pembrolizumab comparisons since significant heterogeneity (I^2^ = 80.65%, p = 0.0064) was determined between KEYNOTE studies), cemiplimab was superior to pembrolizumab, although this superiority was statistically significant only when KEYNOTE-042 results were considered (KEYNOTE-042 [33] (HR 0.67; 95% CI 0.49–0.90; *p* = 0.008); KEYNOTE-024 [13,31] (HR 0.93; 95% CI 0.63–0.36; *p* = 0.621)). Additionally, nivolumab was inferior to pembrolizumab (KEYNOTE-024 [13,31]; (HR 0.47; 95% CI 0.31–0.70; *p* = 0.000); KEYNOTE-042 [33] results; (HR 0.76; 95% CI 0.58–0.99; *p* = 0.04), atezolizumab (HR 0.59; 95% CI 0.39–0.90; *p* = 0.014) and cemiplimab (HR 0.50; 95% CI 0.36–0.71; *p* = 0.001). In the assessment of relative efficacy for PFS, pembrolizumab (KEYNOTE-024 [13,31]) ranked highest followed by cemiplimab and atezolizumab. KEYNOTE-042 [33] results did not confirm pembrolizumab superiority.

In terms of OS, no statistically significant results were determined by indirect comparisons (Table 3). Results from both KEYNOTE studies were grouped for pembrolizumab comparisons since there was no significant heterogeneity between the studies (I^2^ = 0.0%, *p* = 0.6978). In the assessment of relative efficacy for OS, cemiplimab ranked highest followed by atezolizumab, pembrolizumab, durvalumab, and nivolumab. 

### 3.4. Subgroup Analysis

**Figure 3:**
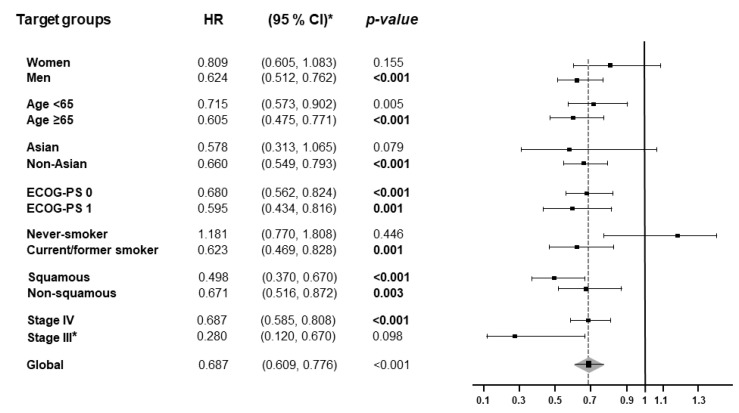
Forest plot of hazard ratios for overall survival (OS) in the subgroup analysis. HR, hazard ratio; CI, confidence interval. * Only KEYNOTE-042 included patients with stage III NSCLC.

OS subgroup analyses were carried out according to sex (women vs. men), age (<65 years vs. ≥65 years), race (Asian vs. non-Asian), Eastern Cooperative Oncology Group performance status (ECOG-PS = 0 vs. ECOG-PS = 1), smoking status (never-smoker vs. current/former smoker), and histology (squamous vs. non-squamous). As shown in Figure 3 and Supplementary Figure S3, overall, first-line PD-(L)1 monotherapy improved OS in almost all subgroups, reaching statistical significance in men (HR_pooled_ = 0.624, 95% CI: 0.51–0.72, *p* < 0.001), non-Asian patients (HR_pooled_ = 0.66, 95% CI: 0.55–0.79, *p* < 0.001), all patients regardless of age (<65 years: HR_pooled_ = 0.72, 95% CI: 0.57–0.90, *p* = 0.005; ≥65 years: HR_pooled_ = 0.61, 95% CI: 0.48–0.77, *p* < 0.001), ECOG PS status (ECOG PS = 0, HR_pooled_ = 0.68, 95% CI: 0.56–0.82, *p* < 0.001; ECOG PS = 1, HR_pooled_ = 0.59, 95% CI: 0.43–0.82, *p* = 0.001), and tumor histological type (Squamous, HR_pooled_ = 0.49, 95% CI: 0.37–0.67, *p* < 0.001; Non-squamous, HR_pooled_ = 0.67, 95% CI: 0.52–0.87, *p* = 0.003). In the case of smokers and NSCLC stage, only current/former smokers (HR_pooled_ = 0.623, 95% CI: 0.47–0.83, *p* = 0.001) benefited from single PD-(L)1 monotherapy over chemotherapy.

### 3.5. Efficacy Results According to Tumor Mutational Burden

OS and PFS were also analyzed according to TMB. A cut-off value of 16 mutations per megabase was established. The number of patients with available TMB results in each study were: 104 patients in KEYNOTE-024; 345 patients in KEYNOTE-042; 107 patients in CheckMate-026 and 87 patients in IMpower110. As shown in Supplementary Figure S4A, in terms of PFS, a benefit with PD-(L)1 monotherapy was observed in patients with TMB ≥16, but not in those with lower cut-off values in all the studies included in the analysis. A similar trend was observed for OS (Supplementary Figure S4B), except for nivolumab (CheckMate-026), for which no benefits were observed in any case. 

## 4. Discussion

The development of ICIs, and specifically of antibodies against programmed death-1 (PD-1) and its ligand (PD-L1), have dramatically altered the therapeutic scenario in NSCLC. The optimal treatment strategy for advanced disease has been the focus of several randomized clinical trials with promising findings that have resulted in the approval of some combined strategies containing PD (L)-1 inhibitors in the first or subsequent lines of treatment [10,56,57,58,59,60,61,62,63]. Despite this success, there are still some patients who do not respond to immune-checkpoint blockade, turning predictive biomarkers have become a useful tool to guide the selection of individuals for these therapies [64]. In this sense, PD-L1 has been identified as a potential good predictive biomarker to select those treatment naïve and refractory patients more likely to respond to immunotherapy [65]. To date, pembrolizumab, and more recently atezolizumab, have received FDA approval as first-line monotherapy in patients with high PD-L1 expression based on the KEYNOTE-024/042 [13,31,32,33] and Impower-110 [34] trial results, respectively. Additionally, cemiplimab, supported by the results of the EMPOWER Lung-01 [37], has been accepted for FDA priority review, and a final decision is expected by February 2021. In this NMA, we evaluated these trials along with others assessing the efficacy of first-line PD (L)-1 monotherapy.

Our results demonstrate an overall benefit in terms of both PFS and OS of PD-(L)1 monotherapy over chemotherapy in advanced NSCLC patients showing high PD-L1 expression. While other MAs have evaluated the efficacy and/or safety of PD-(L)1-containing strategies according to PD-L1 status [66,67,68], this is the first MA to date to include studies evaluating front-line single immunotherapy agents with a PD-L1 enriched design. The latest data available were considered for this NMA, including trials such as the EMPOWER Lung-01, the results of which were recently presented at the ESMO 2020 virtual congress [37]. Although the selection of single-agent immunotherapy or combination immunotherapy for first-line treatment of advanced NSCLC remains controversial among medical oncologists, our results support the evidence that single PD-(L)1 inhibitor monotherapy is beneficial compared to chemotherapy alone in patients with high PD-L1 expression (≥50%). Further studies are required to assess the potential benefit/risk ratio of monotherapy vs. immunotherapy combination strategies. 

It is notable, however, that KEYNOTE-042 was the only study among those analyzed in this NMA that included locally advanced NSCLC patients. These patients usually show better efficacy outcomes given their less advanced stage, which may explain the superiority of pembrolizumab.

For PFS, cemiplimab performed better than pembrolizumab in indirect comparisons, but results were only significant when KEYNOTE-042 [33] data were considered. In addition, nivolumab was inferior to pembrolizumab in both KEYNOTE-024 [13,31] and KEYNOTE-042 [33], and to atezolizumab and cemiplimab. In the assessment of relative efficacy for PFS, pembrolizumab (KEYNOTE-024 [13,31]) again ranked highest, followed by cemiplimab and atezolizumab. Nevertheless, the study population of KEYNOTE-024 was highly selective [13,31] and more importantly, the results were not further replicated in subsequent analyses, such as that included in KEYNOTE-042 [33,55]. This could explain why in our indirect comparisons showed that pembrolizumab results from KEYNOTE-042 [33] ranked lowest compared to the other PD (L)-1 inhibitors. The fact that KEYNOTE-042 was the only trial including locally advanced and advanced NSCLC patients must also be considered when interpreting the results, given the better efficacy outcomes in the locally advanced population (HR 0.28, 95% CI 0.12–0.72 vs. HR 0.75, 95% CI 0.60–0.94). Finally, although cemiplimab results are promising, the median follow-up period was short (10.8 months for cemiplimab) [37], and further studies are required to confirm their efficacy and safety outcomes. 

In the assessment of relative efficacy regarding OS, cemiplimab ranked highest followed by atezolizumab, pembrolizumab, durvalumab, and nivolumab. In terms of OS, no significant results were determined by indirect comparisons. Importantly, it should be noted that OS was not a primary endpoint for either KEYNOTE-024 [13,31,32] or CheckMate-026 [35]. The same considerations previously mentioned for PFS must be taken into account when interpreting these results. 

With respect to subgroup analyses, benefits in OS were reported across the different categories. Patients benefited from first-line anti-PD-(L)1 monotherapy regardless of cancer histology (squamous or non-squamous) or age. Regarding cancer histology no indirect comparisons of efficacy of single anti-PD-L1 agents were performed in squamous versus non squamous lung cancer patients as subgroup analyses were only exploratory in nature. Specifically, the impact of advanced age on the effectiveness of ICIs has not been strongly established so far [69], but older patients are usually more frail, a fact that strengthens the importance of our results. In line with this, OS values according to ECOG PS also showed overall benefits for immunotherapy over chemotherapy. The impact of performance status on the efficacy of immunotherapy is already well known [70]. A recent NMA of real-world data suggests that performance status at treatment initiation retains prognostic significance in patients with immunotherapy, with worse outcomes determined for patients with poorer clinical conditions [71]. However, our study supports the notion that this group of patients with worse conditions may also benefit from first-line immunotherapy monotherapy. Regarding smoking status, the results of our NMA are in line with those obtained in previous studies. Thus, in general, a better response to PD-(L)1 inhibitors was observed in current or former smokers than in non-smokers [72,73,74,75]. Although smoking status is frequently considered an important factor, it should be mentioned that the cemiplimab studies did not include the never-smoker subpopulation. As expected, both histological types benefited equally from treatment. Additionally, it is important to note that, apart from the overall benefits shown by PD-(L)1 inhibitor monotherapy in terms of efficacy, immunotherapy has been found to be associated with lower toxicity compared to chemotherapy [76], which clearly represents an improvement for patients. Additionally, the putative differences between PD-L1 and PD-1 inhibitors should be considered. Thus, as demonstrated by an NMA published in 2019 [7], and according to the available evidence, PD-L1 may have the best safety profile in terms of both treatment-related and immune-related adverse events compared to PD-1 inhibitors. Finally, in the real-world setting, some studies have demonstrated that patients with high PD-L1 expression levels show better response to first-line immunotherapy monotherapy [77,78]. In this regard, EMPOWER Lung-01 is the only study to date to show a correlation between the efficacy improvements achieved in the experimental arm and baseline PD-L1 expression levels [37].

Other clinical factors should be considered when assessing the efficacy of the immunotherapy, such as the presence of liver or brain metastases and the TMB. As demonstrated in our NMA, the latter showed a remarkable predictive value for efficacy, with clear improvements with immunotherapy monotherapy showing improvements in terms of PFS for patients with cut-off values above 16 mutations per megabase, and a similar trend was observed in terms of OS. This is in line with the results obtained with anti-PD-(L)1 regimens in different lines of treatment, which showed better efficacy outcomes for those patients with higher TMB values [27,44,79,80,81,82,83,84].

This NMA has also some limitations. First, the PD-L1 assay methods were not consistent across all studies. Thus, in IM110 [28] Ventana PD-L1 (SP142), an immunohistochemistry assay was used in both TCs and ICs, while in the MYSTIC trial [36], CheckMate-026 [35], and EMPOWER Lung-01, only TCs were considered and differently assessed by Ventana PD-L1 (SP263), Dako PD-L1 22C3, and Dako 28–8, respectively. Moreover, for the KEYNOTE studies [13,31,32,33,55], Dako PD-L1 immunohistochemistry assay 22C3 for TPS was used. Second, the final analysis for both PFS and OS was not available for two studies (EMPOWER Lung-01 [37] and IMpower110 [28]), which may change the overall efficacy in the future. Third, some data were not available, such as PFS results in the MYSTIC trial [36]. Finally, the subgroup analysis relied on limited available information, and results must therefore be interpreted with caution. In this regard, certain limitations were also found in the available EMPOWER Lung-01 data [37], the results of which have thus far only been published as personal communications at conferences. Last, due to the relatively low number of trials involved in this NMA, the results of heterogeneity between studies must be considered as guidance only. Despite these limitations, our results confirm those obtained in individual studies.

In conclusion, first-line anti-PD-(L)1 monotherapy resulted in significantly longer OS and PFS in advanced NSCLC patients with high PD(L)1 expression compared to chemotherapy alone. This supports the potential of this therapeutic option as a first-line strategy for this subgroup of patients. However, efficacy should be further evaluated in comparison with anti-PD-(L)-1-chemotherapy combinations. Additionally, although some drugs yielded significant results in indirect comparisons, the heterogeneity of results support the requirement for further evaluations to determine the superiority of any specific PD-(L)1 inhibitor. 

## Figures and Tables

**Table 1:** Characteristics and main outcomes of the studies included in the meta-analysis (the most up-to-date data have been used for this network meta-analysis).

Study	PD-L1 Expression	Primary Endpoint	Experimental Arm ***	Control Arm ***	Experimental Arm ^#^	Control Arm ^#^	Analysis Timing
KEYNOTE-024 [13,31,32]	High (≥50% of TPS)	PFS (ITT-WT *)	Pembrolizumab (*n* = 154)	Platinum-based chemotherapy (*n* = 151)	Pembrolizumab (*n* = 154)	Platinum-based chemotherapy (*n* = 151)	PFS: Final
EMPOWER Lung-01 [37]	High (≥50% of TCs)	PFS (ITT-WT **) OS (ITT-WT **)	Cemiplimab (*n* = 283)	Platinum-based chemotherapy ^a^ (*n* = 280)	Cemiplimab (*n* = 283)	Platinum-based chemotherapy (*n* = 280)	PFS: Interim OS: Interim
IMpower110 [34]	High (≥50% of TCs or ≥10% ICs) ^a^ High and intermediate (≥5% of TCs or ICs) Any expression level (≥1% of TCs or ICs)	OS (ITT *)	Atezolizumab (*n* = 107)	Platinum-based chemotherapy ^b^ (*n* = 98)	Atezolizumab (*n* = 285)	Platinum-based chemotherapy (*n* = 287)	OS: Interim
KEYNOTE-042 [33,55]	High (≥50% of TPS) ^a^ Intermediate (≥20% of TPS) Low (≥1% of TPS)	OS (ITT-WT *)	Pembrolizumab (*n* = 299)	Platinum-based chemotherapy (*n* = 300)	Pembrolizumab (*n* = 637)	Platinum-based chemotherapy (*n* = 637)	OS: Final
MYSTIC [36]	PD-L1 ≥25% (assessed in TCs) ^b^ PD-L1 <25% (assessed in TCs)	PFS (ITT-WT *) OS (ITT-WT *)	Durvalumab ± tremelimumab ^c^ (*n* = 118)	Platinum-based chemotherapy ^b^ (*n* = 107)	Durvalumab ± tremelimumab (*n* = 369)	Platinum-based chemotherapy (*n* = 352)	PFS: Final OS: Final
CheckMate-026 [35]	PD-L1 ≥5% ^b^ (assessed in TCs) PD-L1 <5% (assessed in TCs)	PFS (ITT-WT *)	Nivolumab (*n* = 88)	Platinum-based chemotherapy (*n* = 126)	Nivolumab (*n* = 271)	Platinum-based chemotherapy (*n* = 270)	PFS: Final

* Patients with EGFR or ALK mutations excluded ** Patients with EGFR, ALK, or ROS1 mutations excluded *** Patients included in this network meta-analysis (high PD-L1 expression (≥50%)) ^#^ Total of patients randomized in each study ^a^ Only the subgroup of patients with PD-L1 ≥50% were included ^b^ Only the durvalumab monotherapy arm was considered for the study PD-L1, programmed cell death-ligand 1; PFS, progression-free survival; OS, overall survival; ITT, intention-to-treat; TCs, tumor cells; ICs, tumor-infiltrating immune cells; EGFR, epidermal growth factor receptor; ALK, anaplastic lymphoma kinase; TPS, tumor proportion score. All studies enriched their populations by selecting patients according to their PD-L1 expression status. In two of them (KEYNOTE-024 [13,35] and EMPOWER Lung-01 [37]), only patients with PD-L1 expression levels ≥50% were included. In the IMpower-110 [28], KEYNOTE-042 [33], and CheckMate-026 [35] studies, patients with PD-L1 expression on at least 1% of TCs or at least 1% of tumor-infiltrating ICs were included and further classified into different groups according to PD-L1 expression level. Finally, in the MYSTIC trial [36], patients were selected regardless of their PD-L1 expression status and subsequently stratified into patients with PD-L1 < 25% and PD-L1 ≥ 25%. In all cases, and in order to compare homogenous populations, only subjects with PD-L1 ≥ 50% were considered for this network meta-analysis (NMA).

**Table 2:** Network meta-analysis: PFS (HR, 95% CI and *p*-values are shown).

	Pembrolizumab (KEYNOTE-024)	Cemiplimab	Atezolizumab	Pembrolizumab (KEYNOTE-042)
**Cemiplimab**	0.93 (0.63–1.36) *p* = 0.621			
**Atezolizumab**	0.79 (0.50–1.26) *p* = 0.317	0.86 (0.57–1.29) *p* = 0.457		
**Pembrolizumab** **KEYNOTE-042**	0.62 (0.43–0.89) *p* = 0.009	0.67 (0.49–0.90) *p* = 0.008	0.78 (0.53–1.15) *p* = 0.204	
**Nivolumab**	0.47 (0.31–0.70) *p* = 0.000	0.50 (0.36–0.71) *p* = 0.001	0.59 (0.39–0.90) *p* = 0.014	0.76 (0.58–0.99) *p* = 0.040

Note: The table must be read as the drug in the column against the drug in the row. For example, the PFS HR of pembrolizumab (KEYNOTE-024) against cemiplimab is 0.93 (95% CI 0.63, 1.36). No results available for durvalumab.

**Table 3:** Network meta-analysis: OS (HR, 95% CI and *p*-values are shown).

	Cemiplimab	Atezolizumab	Pembrolizumab (KN-024/KN-042)	Durvalumab
**Atezolizumab**	0.97 (0.58–1.60) *p* = 0.893			
**Pembrolizumab** **(KN-024/KN-042)**	0.84 (0.59–1.19) *p* = 0.319	0.87 (0.58–1.29) *p* = 0.483		
**Durvalumab**	0.75 (0.48–1.16) *p* = 0.199	0.78 (0.47–1.29) *p* = 0.331	0.90 (0.63–1.30) *p* = 0.578	
**Nivolumab**	0.63 (0.40–1.01) *p* = 0.056	0.66 (0.38–1.12) *p* = 0.123	0.76 (0.51–1.12) *p* = 0.166	0.84 (0.52–1.36) *p* = 0.484

Note: The table must be read as the drug in the column against the drug in the row. For example, the OS HR of cemiplimab against atezolizumab is 0.97 (95% CI 0.58, 1.60). KN, KEYNOTE.

## Data Availability

Qualified researchers may request access to individual patient level data through the clinical study data request platform (https://vivli.org/). Further details on Roche’s criteria for eligible studies are available here (https://vivli.org/members/ourmembers/). For further details on Roche’s Global Policy on the Sharing of Clinical Information and how to request access to related clinical study documents, see here (https://www.roche.com/research_and_development/who_we_are_how_we_work/clinical_trials/our_commitment_to_data_sharing.htm).

## Supplementary Materials

The following are available online at https://www.mdpi.com/article/10.3390/jcm10071365/s1.
