# Supplementary material for: PD-(L)1 Inhibitors as Monotherapy for the First-Line Treatment of Non-Small-Cell Lung Cancer Patients with High PD-L1 Expression: A Network Meta-Analysis
Source: J Clin Med. 2021 Mar 26;10(7):1365. doi: 10.3390/jcm10071365 (PMC8036854; doi:10.3390/jcm10071365)
Supplement: Supplementary file 1 [file jcm-10-01365-s001.pdf]

Supplementary Materials

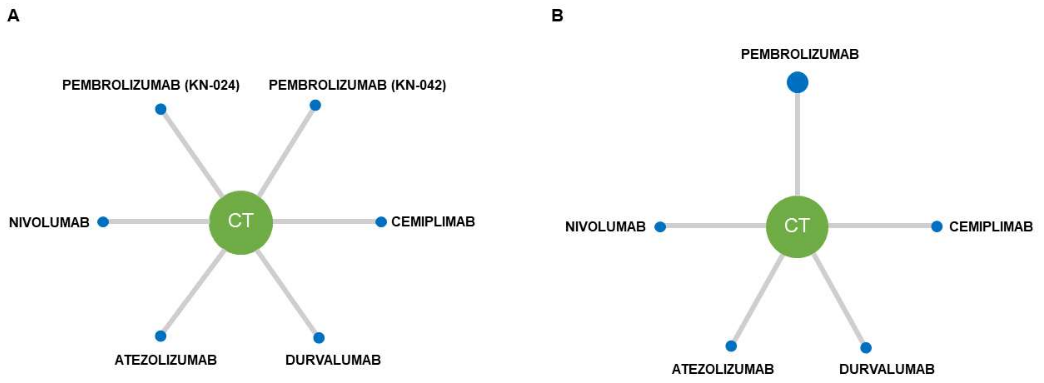

Supplementary Figure 1. Network of studies comparing progression-free survival (PFS) (**A**) and overall survival (OS) (**B**). **CT**, chemotherapy.

Studies	Weight	RR (95% CI)	Treat.	Ctr.
KEYNOTE-024	14.92 %	1.611 (1.180, 2.199)	69/154	42/151
EMPOWER Lung-01	22.44 %	1.927 (1.466, 2.532)	111/283	57/280
IMpower110	17.29 %	1.341 (0.904, 1.990)	41/107	28/98
KEYNOTE-042	23.44 %	1.233 (0.993, 1.532)	118/299	96/300
CheckMate-026	16.92 %	0.784 (0.510, 1.205)	23/88	42/126
Overall ($I^2 = 72.18$ %; $p = 0.006$)		1.354 (1.04, 1.762)	362/931	265/955

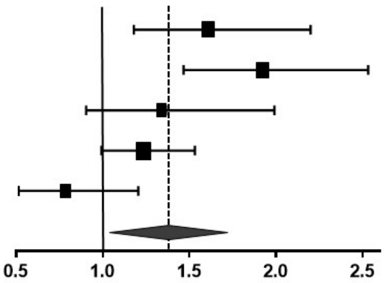

Supplementary Figure 2. Forest plot of pooled odds ratios for overall response rate (ORR) in patients with high PD-L1 expression (≥50%) who received first-line PD-(L)1 monotherapy *vs*. chemotherapy alone. **Treat.**, events in treatment arm/total; **Ctr.**, events in control arm/total; **CI**, confidence interval; **RR**, risk ratio.

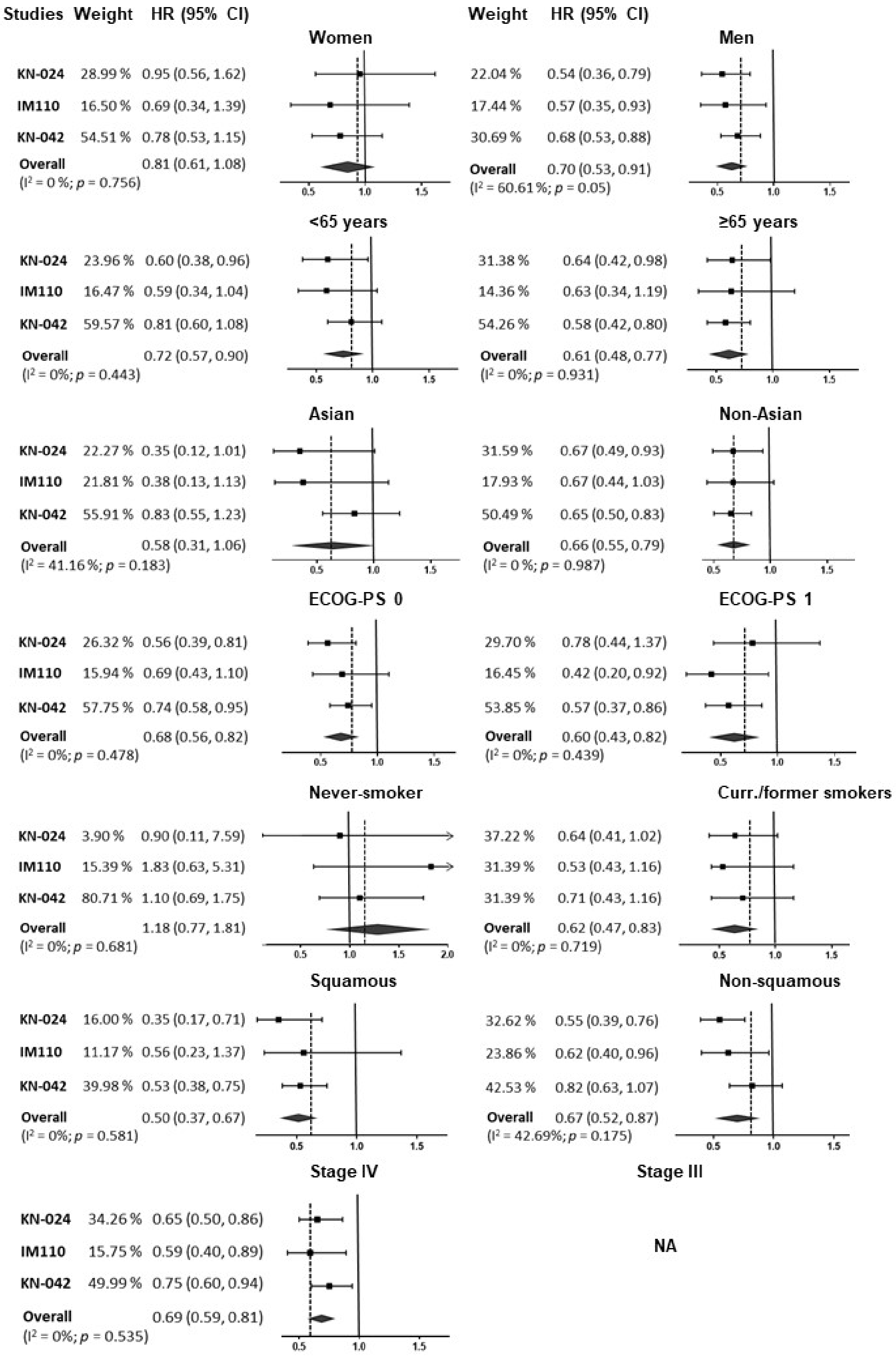

Supplementary Figure 3. Forest plot of hazard ratios for overall survival (OS) in the different patient subgroups. **Curr.,** current; **HR**, hazard ratio; **CI**, confidence interval. **IM**, IMpower; **KN**, KEYNOTE; **CM**, CheckMate; **NA**, not applicable.

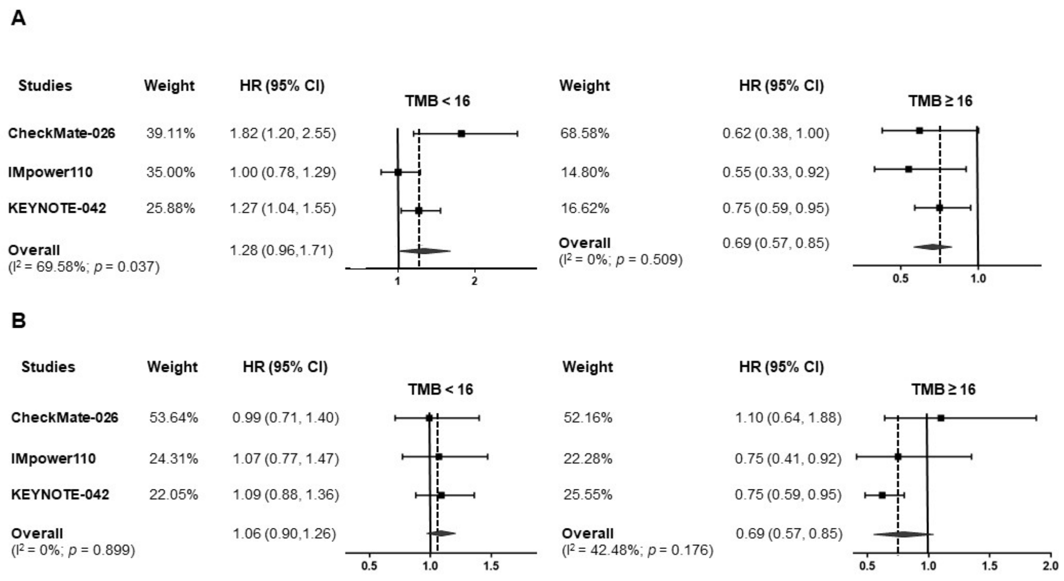

Supplementary Figure S4. Forest plot of hazard ratios for progression-free survival (PFS) (**A**) and overall survival (OS) (**B**) according to TMB. **TMB,** tumour mutational burden; **HR**, hazard ratio; **CI**, confidence interval.

Supplementary Table 1. Characteristics of the patient population (PD-l ≥50%) of the studies included in the meta-analysis.

Study	Experimental arm	Total *n*	Men *n* (%)	Median age (range)	ECOG-PS 0/1 (%)	Never smoker/ current or former smokers (%)	Squamous/non -squamous (%)	Brain metastasis *n* (%)	Previous systemic neoadjuvant therapy *n* (%)	Previous systemic adjuvant therapy *n* (%)
KEYNOTE-024 [10, 33, 34]	Pembrolizumab	154	92 (59.7)	64.5 [33-90]	35.1/64.3	3.20/96.8	18.8/81.2	18 (11.7)	3 (1.9)	6 (3.9)
EMPOWER Lung-01 [39]	Cemiplimab	283	248 (87.6)	63 [31-79]	27.2/72.8	NA	43.1/56.9	34 (12)	3 (1.1)	5 (1.8)
IMpower110 [36]	Atezolizumab	107	79 (73.8)	63 (33–79)	32.7/67.3	8.4/91.6	25.2/74.8	-	-	-
KEYNOTE-042 [35, 57]	Pembrolizumab	299	205 (69)	63 (56–68)	32/68	21/79	36/64	19 (6%)	1 (<1%)	8 (3%)
MYSTIC [38]	Durvalumab	88				14.70/85.30				
CheckMate-026 [37]	Nivolumab	278	184 (68)	63 (32–89)	31/68	11/88	NA	33 (12)		
